# Test–Retest Reliability and Responsiveness of PROMIS Sleep Short Forms Within an RCT in Women With Fibromyalgia

**DOI:** 10.3389/fpain.2021.682072

**Published:** 2021-06-08

**Authors:** Ruth L. Chimenti, Barbara A. Rakel, Dana L. Dailey, Carol G. T. Vance, Miriam B. Zimmerman, Katharine M. Geasland, Jon M. Williams, Leslie J. Crofford, Kathleen A. Sluka

**Affiliations:** ^1^Department of Physical Therapy and Rehabilitation Science, University of Iowa, Iowa City, IA, United States; ^2^College of Nursing, University of Iowa, Iowa City, IA, United States; ^3^Department of Physical Therapy, St. Ambrose University, Davenport, IA, United States; ^4^College of Public Health, University of Iowa, Iowa City, IA, United States; ^5^Department of Medicine/Rheumatology & Immunology, Vanderbilt University, Nashville, TN, United States

**Keywords:** sleep wake disorders, patient reported outcome measures, reproducibility of results, psychometrics, transcutaneous electric nerve stimulation

## Abstract

**Background:** Nonrestorative sleep is commonly reported by individuals with fibromyalgia, but there is limited information on the reliability and responsiveness of self-reported sleep measures in this population.

**Objectives:** (1) Examine the reliability and validity of the Patient-Reported Outcomes Measurement Information System (PROMIS) sleep measures in women with fibromyalgia, and (2) Determine the responsiveness of the PROMIS sleep measures to a daily transcutaneous electrical nerve stimulation (TENS) intervention in women with fibromyalgia over 4 weeks compared with other measures of restorative sleep.

**Methods:** In a double-blinded, dual-site clinical trial, 301 women with fibromyalgia were randomly assigned to utilize either Active-TENS, Placebo-TENS, or No-TENS at home. Measures were collected at baseline and after 4 weeks of treatment. To assess self-reported sleep, the participants completed three PROMIS short forms: Sleep Disturbance, Sleep-Related Impairment, Fatigue, and the Pittsburgh Sleep Quality Index (PSQI). To assess device-measured sleep, actigraphy was used to quantify total sleep time, wake after sleep onset, and sleep efficiency. Linear mixed models were used to examine the effects of treatment, time, and treatment*time interactions.

**Results:** The PROMIS short forms had moderate test–retest reliability (ICC 0.62 to 0.71) and high internal consistency (Cronbach's alpha 0.89 to 0.92). The PROMIS sleep measures [mean change over 4 weeks, 95% confidence interval (CI)], Sleep Disturbance: −1.9 (−3.6 to −0.3), Sleep-Related Impairment: −3 (−4.6 to −1.4), and Fatigue: −2.4 (−3.9 to −0.9) were responsive to improvement in restorative sleep and specific to the Active-TENS group but not in the Placebo-TENS [Sleep Disturbance: −1.3 (−3 to 0.3), Sleep-Related Impairment: −1.2 (−2.8 to 0.4), Fatigue: −1.1 (−2.7 to 0.9)] or No-TENS [Sleep Disturbance: −0.1 (−1.6 to 1.5), Sleep-Related Impairment: −0.2 (−1.7 to 1.4), Fatigue: –.3 (−1.8 to 1.2)] groups. The PSQI was responsive but not specific with improvement detected in both the Active-TENS: −0.9 (−1.7 to −0.1) and Placebo-TENS: −0.9 (−1.7 to 0) groups but not in the No-TENS group: −0.3 (−1.1 to 0.5). Actigraphy was not sensitive to any changes in restorative sleep with Active-TENS [Sleep Efficiency: −1 (−2.8 to 0.9), Total Sleep Time: 3.3 (−19.8 to 26.4)].

**Conclusion:** The PROMIS sleep measures are reliable, valid, and responsive to improvement in restorative sleep in women with fibromyalgia.

**Clinical Trial Registration:** www.ClinicalTrials.gov, identifier: NCT01888640.

## Introduction

Individuals with fibromyalgia commonly report widespread musculoskeletal pain, fatigue, and nonrestorative sleep contributing to limited participation in work and recreational activities (1, 2). There are conflicting findings with nonrestorative sleep detected on self-report questionnaires yet an absence of impairment with device-measured sleep assessment (e.g., accelerometer, polysomnography) in this population (2–5). Conflicting results could be explained in that self-reported and device-measured sleep are different constructs or by limitations in reliability or validity. The Pittsburgh Sleep Quality Index (PSQI) has long been considered a gold standard for self-reported sleep (6). The PSQI has a total score that can distinguish between “good” and “poor” sleepers (7). While the PSQI recognizes multiple dimensions of sleep with seven component scores, these components have not been subsequently validated and are inconsistent with factor analysis (8). Moreover, there may be a floor effect, where the PSQI may not be responsive to treatment in the population with fibromyalgia, where 96% is categorized as “poor” sleepers (9). Together, this underscores the importance of examining the reliability and responsiveness of the PSQI in women with fibromyalgia and other measures that may have superior psychometric properties.

Both the Patient-Reported Outcomes Measurement Information System (PROMIS) sleep disturbance and sleep-related impairment short forms measure a single component of sleep (6, 10). PROMIS questionnaires were developed using item response theory, which could provide greater reliability and validity than legacy questionnaires (10). The reliability and validity of the PROMIS sleep short-forms are established with quantitative and qualitative measures (6, 10–14). However, there remains a lack of information on reliability and responsiveness over a sufficiently long time period when the effectiveness of an intervention may be assessed. Information on reliability, when no treatment is provided, and responsiveness to detect change, when treatment is provided, are needed to determine the interpretation of improvement in sleep measures for individuals with fibromyalgia.

A reciprocal relationship between sleep and pain has been reported by individuals with fibromyalgia and supported by cross-sectional studies (15, 16), yet prospective study designs either fail to detect sleep quality to predict pain (17, 18) or detect small effect sizes (beta = 0.11 to 0.25) (9, 19, 20). We have recently shown that active transcutaneous electrical nerve stimulation (TENS) has reduced resting pain by −1.2 points (0 to 10-point scale, 95% CI: −0.4 to −2.1) compared with Placebo-TENS and by −1.4 points (−0.6 to −2.2) compared with no treatment group (21). The effect of TENS on sleep has not yet been examined for this clinical trial. Given that the lower limit of the 95% CI included small effects (−0.4 to −0.6) of TENS on resting pain, a reliable and responsive measure of restorative sleep may be needed to detect improvement.

The first aim of this study was to examine the reliability and validity of the PROMIS sleep measures in women with fibromyalgia. We hypothesized that the PROMIS short forms would demonstrate superior psychometric properties (reliability, validity) compared with the PSQI and actigraphy in women with fibromyalgia. The second aim was to determine the responsiveness of the PROMIS sleep short forms to daily TENS intervention in women with fibromyalgia compared with the PSQI and actigraphy over a 4-week period. In parallel with previously published findings on the effect of TENS on pain (21) we hypothesized that Active-TENS would have a greater effect on restorative sleep than the use of Placebo-TENS or No-TENS, and that this effect would be best detected by the PROMIS sleep measures.

## Materials and Methods

### Study Design

**Figure 1:**
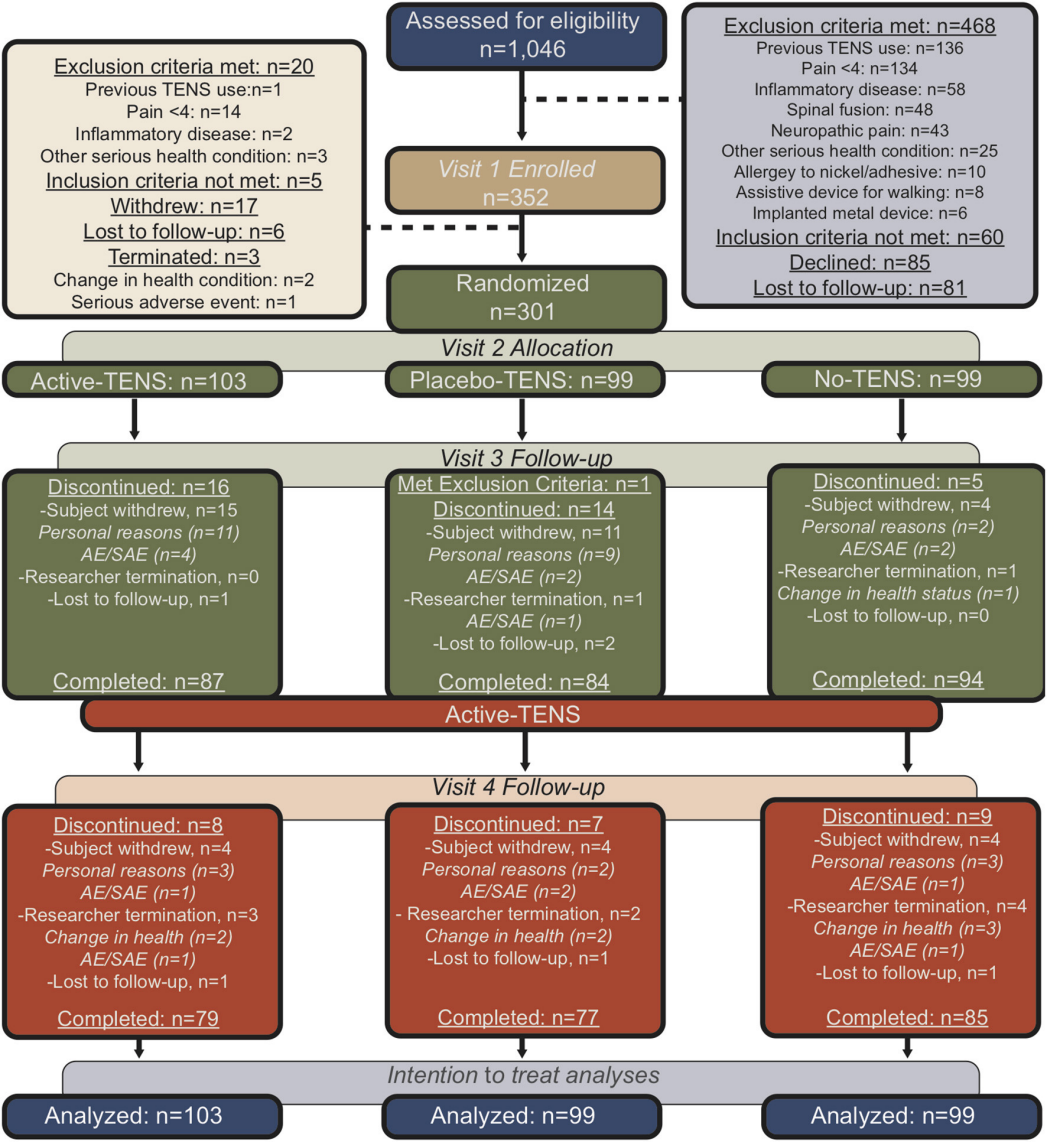
Flow diagram for participants in the trial using the Consolidated Standards of Reporting Trials guidelines.

This is a secondary analysis of data collected during a phase II randomized, double-blinded, placebo-controlled dual-site clinical trial. The Fibromyalgia Activity Study with TENS (FAST) was conducted at two university medical centers (NCT01888640) (21). The participants completed four visits over a 9-week period (Figure 1) (22). The first visit was for in-person screening and consent. The participants had to be diagnosed with fibromyalgia by a physician and meet the 1990 American College of Rheumatology (ACR) criteria for fibromyalgia. Additional inclusion criteria were women, aged 18–70 years, who reported stable medication use for the last 4 weeks and projected stable treatment for the next 2 months. Exclusion criteria were: TENS use in the last 5 years; pain rating of <4/10 on the verbal numerical pain rating scale; serious or unstable medical or psychiatric condition that would preclude participation in the study (surgery in the past or upcoming 3 months, undergoing diagnostic testing for an undiagnosed condition, instability of a condition that is not part of the eligibility criteria); pacemaker; neuropathic or autoimmune disorder; spinal fusion or metal implants in the spine; allergy to adhesive or nickel; pregnancy; epilepsy; and inability to walk 6 min without assistance. At 0 weeks, the participants completed a second screening for eligibility and began the randomized treatment for 4 weeks. The primary endpoint of 4 weeks was chosen; because clinically, physical therapists re-evaluate their patients every 30 days for insurance to document progress and determine if a change in the plan of care is needed. At 4 weeks, all the participants were unblinded and were provided with an Active-TENS unit. At eight-weeks, the final outcome assessment was carried out.

### Participants

The participants were recruited from university clinics and surrounding communities using mass email, posting flyers, and referring clinicians (21). They were provided free parking, mileage reimbursement, and payment for time (21). All the participants continued current treatments prescribed by their healthcare provider and were told not to change medications during the study. All of them provided written informed consent to participate in this study, which was approved by the institutional review boards of both universities.

### Randomization

Between September 2013 and February 2018, 1,046 women were assessed for eligibility, 352 participants were enrolled, and 301 were randomly assigned to one of three groups: Active-TENS (*n* = 103), Placebo-TENS (*n* = 99), or No-TENS (*n* = 99; Figure 1). Randomization was stratified by geographical location and opioid status (opioid vs. non-opioid). The participants were classified as opioid users if they had taken an opioid at least 5/7 days per week for the last 30 days. Randomization was then performed separately within each stratum using permuted block randomization with blocks of six and nine.

### Blinding

To blind the outcome assessors, a mock TENS unit was used in the No-Treatment group during evaluation visits. The participants had a study bag to hide their study materials, and they were asked to not discuss their treatment with the outcome assessor. To blind the participants, only TENS-naïve individuals were eligible to participate and a Placebo-TENS unit was provided. In addition, a nearly identical script was used for patient education, except for a line that differed between the Active-TENS and Placebo-TENS groups. The Active-TENS group was asked to indicate when they could first feel the TENS and then when the intensity was a strong, comfortable, non-painful sensation. In contrast, the Placebo-TENS group was only asked when they could first feel the TENS, and this was the maximum TENS intensity that they were instructed to use when setting up the unit throughout the 4 weeks. Both groups were informed that as the treatment continues they may experience no change or an increase or decrease in the way the TENS feels. This accounted for the lack of sensation experienced in the Placebo-TENS group throughout the remainder of the session. At 4 weeks, the outcome assessors and participants were asked to guess the treatment group allocation.

### Transcutaneous Electrical Nerve Stimulation Intervention

An EMPI-Select TENS (DJO Global, Vista, CA, United States) unit was used to deliver treatment through butterfly electrodes placed on the upper and lower back. To be appropriate for non-opioid and opioid users, the TENS parameters alternated between low and high frequencies (2 to 125 Hz) to activate not only μ-opioid receptors targeted by pharmaceuticals but also δ-opioid receptors (23–27). The intensity was set to strong and comfortable (28). The Placebo-TENS unit delivered current for 45 s ramping down to 0 Hz in the last 15 s and remained at 0 Hz for the remainder of the session (29). The participants were instructed to use TENS at least 2 h per day during activity at home for 4 weeks. They were provided education that TENS was most effective when combined with exercise. Therefore, they were encouraged to use TENS to enhance their ability to complete moderate to vigorous activities. The participants were discouraged from using TENS as a passive treatment during rest and during sleep for safety. The TENS unit collected information on a range of usage, such as number of sessions, average duration of session, and intensity.

### Outcome Measures

#### Demographics and Potential Covariates

Participant characteristics listed in Table 1 were collected at 0 weeks. Disease impact was measured with the Fibromyalgia Impact Questionnaire-Revised (FIQR) (30).

**Table 1:** Demographic and baseline characteristics of study participants.

	**Active-TENS**	**Placebo-TENS**	**No-TENS**	***p*-value**
	***n* = 103**	***n* = 99**	***n* = 99**	
**Demographic variables**				
Age (yrs)	44.7 (14.3)	47.2 (12.6)	48.6 (11.8)	0.10
Body mass index (kg/m^2^)	34.8 (8.7)	33.7 (8.8)	34.0 (8.9)	0.65
Married or Living with partner	34 (33%)	50 (51%)	51 (52%)	**0.010**
Duration of FM (yrs)	7 [3–12]	7 [2–14]	7 [4-15]	0.47
Opioids for pain (#, % yes)	27 (26%)	26 (26%)	26 (26%)	–
**Baseline measures**				
Pain at rest (NRS)	6.2 (1.5)	5.9 (1.4)	6.1 (1.6)	0.33
Fatigue at rest (NRS)	6.8 (2.0)	6.1 (1.8)	6.4 (2.0)	0.08
FIQR- Global score	59.2 (16.8)	53.7 (15.9)	55.6 (16.0)	**0.049**
PROMIS-Anxiety	58.8 (8.7)	58.1 (8.0)	58.3 (7.8)	0.82
PROMIS-Depression	58.1 (8.1)	55.7 (8.5)	56.6 (8.1)	0.12
**TENS use over 4-weeks (randomized, blinded, 0–4-weeks)**				
Intensity Lumbar (mA)	38.1 (8.3)	–	–	–
Intensity Cervical (mA)	38.3 (7.3)	–	–	–
Number of sessions	25.0 [17.0–34.3]	23.0 [12.0–34.5]	–	0.36
Ave duration of session (h)	1.7 [1.2–2.1]	1.8 [1.3–2.3]	–	0.70
Total time (h)	45.0 [27.0–64.0]	42.0 [23.0–60.0]		0.33
**TENS use over 2**^**nd**^ **month (all Active-TENS, unblinded, 4-weeks to 8-weeks)**				
Intensity Lumbar (mA)	41.8 (9.3)	38.3 (7.6)	41.3 (7.6)	**0.011**
Intensity Cervical (mA)	41.3 (8.7)	38.7 (6.7)	41.4 (7.0)	**0.033**
Number of sessions	19.0 [8.0–27.0]	23.5 [15.0–36.0]	26.0 [15.0–34.0]	**0.002**
Ave duration of session (h)	1.8 [ 1.4–2.4]	1.8 [1.3–2.5]	1.8 [1.3–2.5]	0.76
Total time used (h)	45.0 [ 27.0–64.0]	43.0 [21.5–76.3]	45.0 [30.0–71.0]	0.14

*Data presented as mean (standard deviation), median (interquartile range), or number (% of sample) as appropriate*.

*NRS, numeric rating scale; FIQR, fibromyalgia impact questionnaire – revised; PROMIS; patient-reported outcomes measurement information system; FM, fibromyalgia. Statistically significant p-values are in bold*.

#### Patient-Reported Outcomes

Restorative sleep was examined using three questionnaires: the PROMIS Sleep Disturbance (short form 8b, range 28.9–76.5) (10), PROMIS Sleep-Related Impairment (short form 8a, range 30–80) (10), and the PSQI (range 0–21) (7, 9). Fatigue was examined using the PROMIS Fatigue (short form 7a, range 29.4–83.2) (14). For these PROMIS short forms, a t-score of 60 represents 1 SD worse than the general population mean t-score of 50. While the minimally important difference (MID) has not been established for all PROMIS measures, the MID for fatigue is 3 to 5 points for cancer patients (31).

#### Actigraph-Measured Outcomes

Accelerometry (ActiSleep+, ActiGraph, Pensacola, FL, United States) was used to measure sleep. The participants wore the accelerometer on their nondominant wrist for 7 days prior to each time point with 0 weeks indicating sleep during the week prior to baseline measures and 4 weeks indicating sleep during the fourth week of study participation. To interpret actigraph sleep data, the participants kept a log of when they went to bed and got up each day. Validation of reported times was performed using the actigraph data. For “In Bed,” if a spike in activity (count ≥ 1,000 for a period of 5 min or longer) occurred within the stated bedtime, then the bedtime was adjusted to the beginning of the next consecutive 15 min of inactivity (32). For “Out of Bed,” if a spike in activity (count ≥ 1000 for a period of 5 min or longer) was within the stated bedtime, then the “Out of Bed” time was adjusted to indicate it was prior to the spike in activity. Three actigraph-measured sleep variables were chosen for analysis total sleep time, sleep efficiency, and wake after sleep onset (33). The actigraph-measured sleep variables have demonstrated concurrent validity with the gold standard of polysomnography (32, 33). The Cole–Kripke algorithm was used to distinguish “Sleep Onset” from wakefulness after the “In bed” time (32). Total Sleep Time (TST) was defined as the number of minutes between “Sleep Onset” and time “Out of Bed.” Sleep efficiency was defined as TST as a percentage of the time in bed (time difference between “In Bed” and “Out of Bed”). Wake After Sleep Onset (WASO) was defined as the number of minutes awake between “Sleep Onset” and “Out of Bed.”

### Statistical Analysis

Baseline characteristics were compared between groups using one-way ANOVA for continuous variables and Pearson's Chi-squared test for categorical variables. To examine test–retest reliability, we examined the intra-class correlation (ICC) over the 4-week period when no treatment was administered within the No-TENS group. Interpretation of the ICC estimates was based on >0.75 indicating good to excellent reliability and 0.5 to 0.75 indicating moderate reliability (34). Since reliability is a prerequisite for validity, measures with poor test–retest reliability (ICC < 0.50) in the TENS group were not used in subsequent analyses (34). For internal consistency, we examined the inter-item correlations and Cronbach's alpha for the total questionnaire. We also reported the potential improvement in the inter-item correlation if a specific item was deleted to evaluate the impact of each item on the Cronbach's alpha for the total questionnaire. To examine convergent validity, we examined the Pearson correlations between patient-reported outcomes.

A Linear mixed model for repeated measures (0, 4, and 8 weeks) was used to examine (1) the effect of time within each group (0 to 4 weeks and 0 to 8 weeks); (2) the effect of treatment*time interaction from 0 to 4 weeks (SAS version 9.4, SAS/STAT 14.3). To correct for multiple comparisons, Bonferroni adjustment was applied for time (adjusted for six tests) and treatment*time (adjusted for three tests). In addition to the *p*-values, estimates of mean change or mean difference between groups with adjusted 95% CIs were computed. Differences between groups at baseline were entered as covariates of the model. When there was a significant interaction between group and treatment, further analyses examined if this effect was moderated by opioid use. In fitting the linear mixed model, the Akaike Information Criteria (AIC) and Schwarz's Bayesian Information Criteria (BIC) were used to select the covariance structure that best fit these longitudinal measures within the subject. The covariance types that were considered included compound symmetry (CS), heterogeneous CS, first order autoregressive (AR1), and unstructured. From these model parameter estimates and the fitted covariance structure, tests of mean contrast were performed to assess the effect of Active-TENS, compared with Placebo-TENS and No-TENS on sleep outcomes. Statistical significance was defined as *p* < 0.05, and corresponding 95% CIs are provided for all data.

The linear mixed model performed an intent-to-treat analysis, which included all available data from all the randomized study subjects. Four weeks was the primary endpoint with completion rates ranging from 84 to 95% between groups and 8 weeks completion rates ranged from 90 to 92% (Figure 1). Comparison of the 25 participants that dropped out at 8 weeks with those that had completed follow-up through 8 weeks showed no difference in baseline FIQR, PSQI, and PROMIS short forms, or actigraph-measured sleep. The lack of difference in the sleep measures between those who dropped out and those who did not supports the use of the missing at random statistical assumption, which allows for estimation of the sleep measures based on other variables in the dataset (35). The use of likelihood-based methods (i.e., linear mixed model analysis) for analysis of unbalanced data (with missing values) under the missing at random assumption provides valid estimates for inference. As a *post-hoc* analysis, we examined the Pearson correlations between changes in sleep with resting pain and duration of TENS use.

## Results

### Test–Retest Reliability

The PROMIS sleep measures had moderate test–retest reliability in the No-TENS group over a 4-week period with ICCs ranging from.62 to.71 (Table 2). The PSQI global score also had moderate test–retest reliability of.59 (Table 2). The actigraph measures of sleep efficiency, total sleep time, and wake after sleep onset had moderate reliability with ICCs ranging from 0.59 to 0.68.

**Table 2:** Test–retest reliability of sleep measures for the No-TENS group between 0-week and 4-weeks.

		**ICC**	**95% CI**	***p*-value**
PROMIS short-forms	Sleep disturbance (8b)	0.71	0.59 to 0.80	**<0.001**
	Sleep impairment (8a)	0.62	0.47 to 0.73	**<0.001**
	Fatigue (7a)	0.64	0.50 to 0.74	**<0.001**
PSQI	Global score	0.59	0.44 to 0.71	**<0.001**
Actigraph-measured	Efficiency	0.68	0.50 to 0.79	**<0.001**
	Total sleep time	0.59	0.37 to 0.74	**<0.001**
	Wake after sleep onset	0.61	0.40 to 0.75	**<0.001**

*Interpretation of ICC estimates was based on <0.5 indicating poor reliability, 0.5 to 0.75 indicating moderate reliability, and >0.75 indicating good to excellent reliability (34). Statistically significant p-values are in bold*.

### Internal Consistency

The PROMIS short forms of Sleep Disturbance and Sleep-Related Impairment had high internal consistency with Cronbach's alpha of 0.92 and 0.89, respectively (Tables 3A, B). The PSQI had low internal consistency with a Cronbach's alpha of.54 (Table 3C). The Cronbach's alpha would increase to 0.66 if component 6 (use of sleeping medication) was deleted, but even with this deletion, the internal consistency would still not reach an acceptable level (0.7).

**Table 3A:** Inter-item correlations for PROMIS Sleep Disturbance with the questions numbered D1 through D8.

	**D1**	**D2**	**D3**	**D4**	**D5**	**D6**	**D7**	**D8**	**Cronbach alpha if item deleted**
**D1**	1.00								0.90
**D2**	0.71	1.00							0.90
**D3**	0.59	0.66	1.00						0.91
**D4**	0.56	0.58	0.42	1.00					0.92
**D5**	0.63	0.65	0.50	0.45	1.00				0.91
**D6**	0.69	0.71	0.56	0.61	0.77	1.00			0.90
**D7**	0.56	0.60	0.61	0.41	0.49	0.54	1.00		0.91
**D8**	0.59	0.70	0.65	0.49	0.56	0.65	0.64	1.00	0.90

*Cronbach's alpha for the total questionnaire was 0.92 (95% CI: 0.9 to 0.93), and deletion of an item would not improve the reliability of this measure, as seen in the last column*.

**Table 3B:** Inter-item correlations for PROMIS Sleep-Related Impairment with the questions numbered I1 through I8.

	**I1**	**I2**	**I3**	**I4**	**I5**	**I6**	**I7**	**I8**	**Cronbach alpha if item deleted**
**I1**	1.00								0.88
**I2**	0.36	1.00							0.89
**I3**	0.49	0.45	1.00						0.88
**I4**	0.57	0.45	0.53	1.00					0.87
**I5**	0.55	0.40	0.51	0.80	1.00				0.87
**I6**	0.46	0.39	0.42	0.64	0.67	1.00			0.88
**I7**	0.64	0.37	0.50	0.55	0.54	0.52	1.00		0.87
**I8**	0.61	0.25	0.40	0.52	0.50	0.42	0.71	1.00	0.88

*Cronbach's alpha for the total questionnaire was 0.89 (95% CI: 0.87 to 0.91), and deletion of an item would not improve the reliability of this measure, as seen in the last column*.

**Table 3C:** Inter-item correlations for PSQI with the components numbered C1 through C7.

	**C1**	**C2**	**C3**	**C4**	**C5**	**C6**	**C7**		**Cronbach alpha if item deleted**
**C1**	1.00								0.30
**C2**	0.39	1.00							0.39
**C3**	0.44	0.17	1.00						0.37
**C4**	0.33	0.32	0.58	1.00					0.32
**C5**	0.33	0.23	0.16	0.19	1.00				0.41
**C6**	−0.01	0.02	−0.12	0.02	0.02	1.00			0.58
**C7**	0.06	−0.08	−0.05	0.00	0.27	0.05	1.00		0.46

*Cronbach's alpha for the total questionnaire was 0.45 (95% CI: 0.26 to 0.61). Deletion of component 6 (use of sleeping medication) would increase the Cronbach's alpha to 0.58 (95% CI: 0.44 to 0.7) in this sample*.

### Convergent Validity

PROMIS Sleep Disturbance was most strongly correlated with the PSQI (*r* = 0.71), moderately correlated with both PROMIS Sleep-Related Impairment (*r* = 0.5) and PROMIS Fatigue (*r* = 0.33), and minimally correlated with actigraph-measured sleep (*r* < 0.2, Table 4). PROMIS Sleep-Related Impairment was most strongly correlated with PROMIS Fatigue (*r* = 0.63), moderately correlated with both PROMIS Sleep Disturbance (*r* = 0.5) and PSQI (*r* = 0.47), and minimally correlated with actigraph-measured sleep (*r* < 0.1). Actigraph-measured sleep efficiency and wake after sleep onset were strongly correlated (*r* = −0.94), but all other correlations between actigraph measures were weak (*r* = 0.07 to 0.2).

**Table 4:** Convergent validity between PROMIS short forms, Pittsburg Sleep Quality Index, and Actigraph-measured sleep (efficiency, total sleep time, and wake after sleep onset).

	**PROMIS**		**PSQI**		**Actigraph**	
	**Sleep disturbance**	**SRI**	**Fatigue**	**Global**	**Efficiency**	**TST**
Sleep-related impairment (SRI)	***r*** **= 0.50**, *p* < 0.001	1				
Fatigue	***r*** **= 0.33**, *p* < 0.001	***r*** **= 0.63**, *p* < 0.001	1			
PSQI	***r*** **= 0.71**, *p* < 0.001	***r*** **= 0.47**, *p* < 0.001	***r*** **= 0.38**, *p* < 0.001	1		
Efficiency	***r*** **= −0.19**, *p* < 0.001	*r* = −0.02, *p* = 0.76	*r* = −0.03, *p* = 0.65	*r* = −0.27, *p* = 0.65	1	
Total sleep time (TST)	***r*** **= −0.14**, *p* = 0.020	*r* = −0.05, *p* = 0.38	*r* = 0.00, *p* = 0.94	*r* = −0.06, *p* = 0.35	***r*** **= 0.20**, *p* < 0.001	1
Wake after sleep onset (WASO)	***r*** **= 0.15**, *p* < 0.001	*r* = 0.02, *p* = 0.74	*r* = 0.05, *p* = 0.42	***r*** **= 0.29**, *p* < 0.001	***r*** **= −0.94**, *p* < 0.001	*r* = 0.07, *p* = 0.200

*Statistically significant correlations are written in bold*.

### Responsiveness of Sleep Measures During Randomized, Blinded TENS Treatment

**Figure 2:**
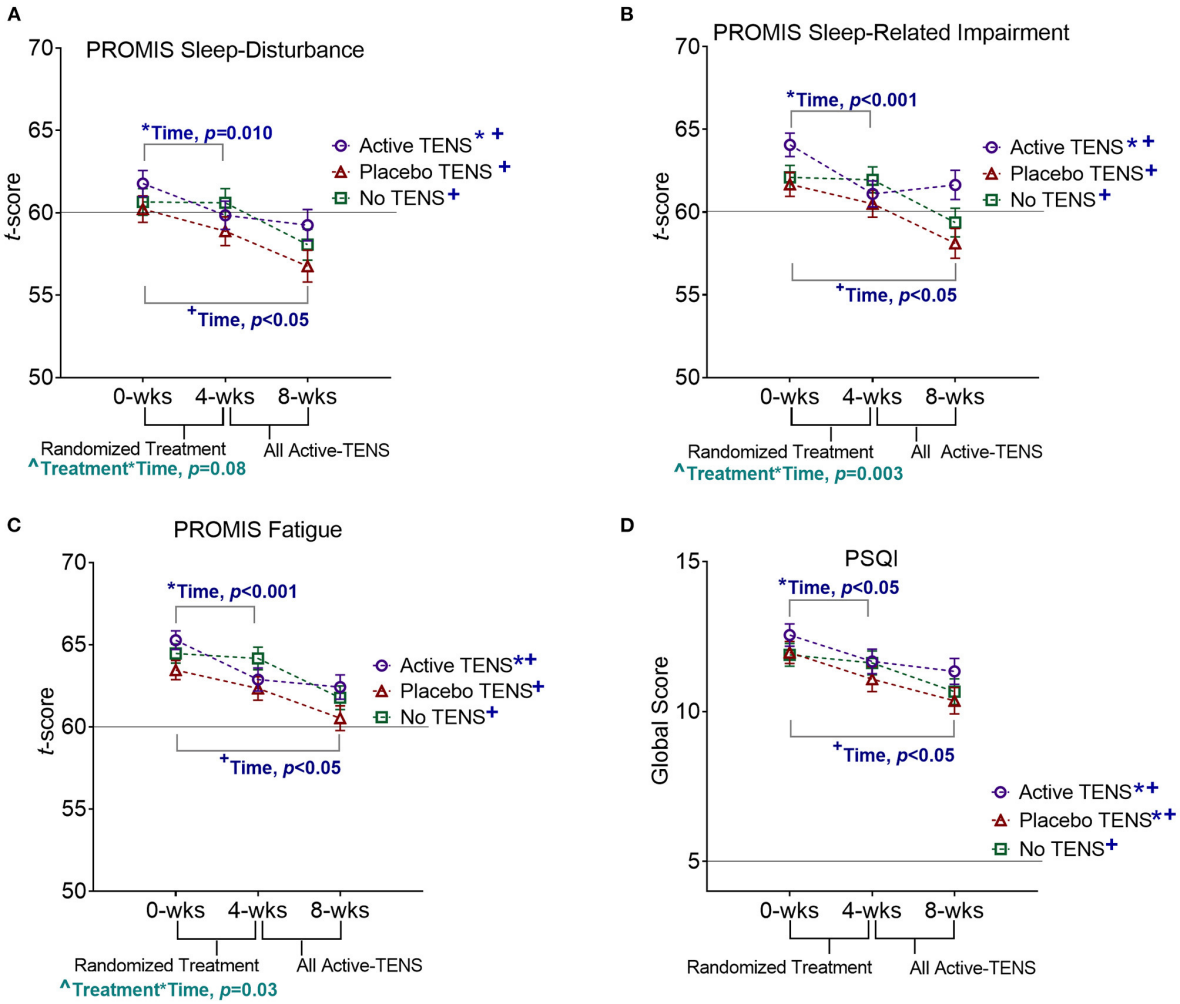
Effects of TENS on **(A)** PROMIS Sleep Disturbance, **(B)** Pittsburg Sleep Quality Index (PSQI), **(C)** PROMIS Sleep-Related Impairment, and **(D)** PROMIS Fatigue. Statistically significant effects of time (*0 to 4 weeks, and ^+^0 to 8 weeks) and interaction effects of treatment*time(∧) are indicated. For all outcome measures, scores higher than the horizontal line are indicative of nonrestorative sleep.

Potential covariates were similar between groups, except that the Active-TENS group had a lower rate of being married/living with a partner (*p* = 0.01) and a higher FIQR score (*p* = 0.049) compared with the other two groups (Table 1). There were no differences between groups in self-reported measures (PROMIS and PSQI) at baseline, except that the Active-TENS group had a higher level of PROMIS sleep-related impairment than the Placebo-TENS group (*p* = 0.04, Supplementary Table 1). From 0 to 4 weeks, the PROMIS short-forms were responsive to improvement in restorative sleep and specific to the Active-TENS group but not to Placebo-TENS or No-TENS (Figure 2). The Active-TENS group reported reduced Sleep Disturbance [mean change (95% CI), −1.9 (−3.6 to −0.3) and Sleep-Related Impairment: −3 (−4.6 to −1.4)] at 4 weeks compared with baseline (Supplementary Table 1). In contrast, the PROMIS sleep measures did not significantly improve for the Placebo-TENS Sleep Disturbance: −1.3 (−3 to 0.3); Sleep-Related Impairment: −1.2 (−2.8 to 0.4) and No-TENS Sleep Disturbance: −0.1 (−1.6 to 1.5); Sleep-Related Impairment: −0.2 (−1.7 to 1.4) groups.

To further examine the significant interaction between treatment group and time for PROMIS Sleep-Related Impairment and Fatigue short forms, we examined the correlations of these variables with resting pain and duration of TENS use. A greater decrease in resting pain was correlated with a greater reduction in Sleep-Related Impairment [*r* = 0.21 (95% CI: 0.09 to 0.32)] and Fatigue [*r* = 0.27 (95% CI: 0.16 to 0.38)]. The significant interactions between treatment group and time became non-significant when resting pain was included in the model (PROMIS Sleep-Related Impairment: *p* = 0.136, PROMIS Fatigue: *p* = 0.505, Supplementary Table 1). More minutes per day of TENS use was associated with greater improvement in restorative sleep (PROMIS Sleep-Related Impairment: *r* = −0.21 (−0.09 to −0.33); PROMIS Fatigue: *r* = −0.17 (−0.05 to −0.28) when accounting for resting pain at baseline.

The PSQI was responsive but not specific with improvement detected in both the Active-TENS (*p* = 0.03) and Placebo-TENS (*p* = 0.04) groups but not in the No-TENS group (*p* > 0.99, Figure 2, Supplementary Table 2). Actigraphy measures were not sensitive to changes in restorative sleep (*p* > 0.05 for all measures). There were no significant effects of visit or group*visit for the PSQI or actigraphy measures (*p* > 0.05). The addition of marital status and baseline FIQR as co-variates in the analysis did not change the significance of these findings (Supplementary Table 3).

### Responsiveness of Sleep Measures During Non-randomized, Unblinded Active-Transcutaneous Electrical Nerve Stimulation Treatment

From 4 to 8 weeks, the Placebo-TENS and No-TENS groups had a significant improvement in the PROMIS short forms and the PSQI during the Active-TENS treatment (*p* < 0.05 for all effects of time at 8 weeks), but there were no changes in actigraph measures (Figure 2, Supplementary Tables 1, 2). The Active-TENS group also had a significant improvement in all self-report measures (time, *p* < 0.05), indicating sustained improvement with additional 1-month use.

#### Integrity of Blinding

The outcome assessor was adequately blinded and correctly guessed treatment allocation for 45% of the Active-TENS group, 13% of the Placebo-TENS group, and 20% of the No-TENS group (21). Given a 50% chance of receiving an Active-TENS or Placebo-TENS unit for home use, the Placebo-TENS group was adequately blinded with 49% of the participants correctly guessing their treatment group, but 70% of the Active-TENS group was able to correctly guess group allocation (21). For the sleep measures that differed between the Active-TENS and Placebo-TENS groups, these differences became non-significant among those who incorrectly guessed group allocation [PROMIS Sleep-Related Impairment: 1.37 (−1.8 to 4.59), *p* = 0.97; PROMIS Fatigue: 1.2 (−1.9 to 4.4), *p* = 0.377)]. In contrast for participants in the Active-TENS and Placebo-TENS groups who correctly guessed their allocation, the difference remained significant [PROMIS Sleep-Related Impairment: −3.7 (95% CI: −6.21 to −1.3, *p* < 0.001), PROMIS Fatigue: −2.8 (−5.3 to −0.4), *p* < 0.001].

### Data Availability Statement

The data that support the findings of this study are available from the corresponding author (RLC) upon reasonable request.

## Discussion

The PROMIS short forms were reliable, valid, and responsive to change during randomized, blinded treatment in women with fibromyalgia. TENS improved self-reported sleep with the PROMIS short forms but not with the PSQI or actigraph measures after 4 weeks of daily use in women with fibromyalgia. The interpretation of the findings, in which improvements in sleep were detected with some measures but not with others, may be related to the relative psychometric properties of the outcome measures.

### Patient-Reported Outcomes Measurement Information System Reliability

The test–retest reliability of all self-reported and actigraph-measured sleep measures was moderate (ICC 0.59 to 0.71), which likely reflects natural fluctuations in sleep over a 4-week period. The test–retest reliability results are within the range of another study that used polysomnography and that reported moderate test–retest reliability at 1 month (sleep efficiency, *r* = 0.52) in individuals with suspected sleep apnea (36). A previous study that validated the PSQI had found good reliability (*r* = 0.85) (7), but it was noted that reliability was lower for participants with depression (PSQI sub-components *r* = 0.19 to 0.83). Similarly, the test–retest reliability of the PROMIS sleep short forms in individuals with rheumatoid arthritis was reported as excellent (*r* = 0.73 to 0.83) (37). The lower reliability in this study may be due to a longer follow-up (2 days vs. 1 month), higher level of depression (49.1 ± 8.8 vs. 56.8 + 8.2), and the limitation of a smaller sample size (*N* = 188 vs. *N* = 99 for the test–retest reliability in the No-TENS group) (21, 37). Since perceptions of nonrestorative sleep, fatigue, and psychological factors are intertwined within an individual and change over time, it may be informative to evaluate changes in sleep within the context of changes in other psychological factors, such as depression.

Consistent with the literature (10, 12, 13, 37), the internal consistency was high for all PROMIS sleep measures (Cronbach's alpha = 0.89 to.91). In contrast, we found the internal consistency of the PSQI to be below the acceptable level of.7 (Cronbach's alpha = 0.54). Published internal consistency measures for the PSQI have varied in the literature from 0.64 to 0.83, and no published studies have reached the level of high internal consistency (Cronbach's alpha ≥0.9) observed with the PROMIS sleep short forms (38). The lower internal consistency of the PSQI is likely due to its design. The PSQI has 18 items that assess seven components of sleep over a 4-week recall period, whereas both the PROMIS Sleep Disturbance and Sleep-Related Impairment have eight items that are focused on a single component of sleep over a 1-week recall period. The higher reliability, supported by differences in internal consistency, of the PROMIS short forms in the sample, potentially allows for greater precision and ability to detect change over time compared with the PSQI.

### Patient-Reported Outcomes Measurement Information System Concurrent Validity

PROMIS Sleep Disturbance was most strongly correlated with the PSQI (*r* = 0.71), which is consistent with the literature validating these measures in other populations (*r* = 0.83 to 0.85) (6, 10). Given that half of the items on the PSQI (9/18) assess sleep disturbance, it is not surprising that variance in the global PSQI score appears to be primarily driven by sleep disturbance items. PROMIS Sleep-Related Impairment had the strongest correlation with PROMIS Fatigue (*r* = 0.63), indicating some overlap between these measures in women with fibromyalgia. Correlations between the PROMIS and actigraph-measured sleep were < 0.2, which is similar to previous studies of individuals with fibromyalgia (4, 5). Okifuji et al. (4) found a nearly equal distribution of participants who overestimated their sleep as those who underestimated sleep with self-report (sleep duration, number of awakenings, and feeling refreshed upon awakening) compared with actigraph-based measures. The lack of correspondence between self-report and device-measured sleep in women with fibromyalgia likely indicates that a problem in an aspect of sleep/wakefulness is not reflective of a problem in other aspects.

### Responsiveness of Patient-Reported Outcomes Measurement Information System Short Forms to Transcutaneous Electrical Nerve Stimulation

There have been no previously published studies of the effects of TENS on restorative sleep. We have previously reported that daily use of TENS with activity reduces pain in women with fibromyalgia at 4 weeks and that this effect was maintained at 8 weeks (21, 39). The findings of this study on sleep follow a similar pattern of improvement as pain. Given the correlations between pain and sleep, the reduced effect of TENS on sleep when pain was included in the model may be due to the mediation effect. This could be further explored in future studies examining the mediation effect of pain on changes in sleep. Comparing the different self-reported sleep measures in this study, the PSQI detected an improvement at 4 weeks in both the Active-TENS and Placebo-TENS groups, indicating a non-specific effect on sleep. In contrast, the PROMIS measures detected improvement at 4 weeks only in the Active-TENS group, indicating improvement in a specific component of restorative sleep. These findings on responsiveness combined with the higher reliability of the PROMIS measures support the validity of these measures in women with fibromyalgia.

### Clinical Relevance

Accurately identifying the factors contributing to nonrestorative sleep can direct treatment decisions for fibromyalgia symptoms, which include both pharmacological (e.g., antidepressants) and non-pharmacological (e.g., cognitive behavioral therapy) options (40, 41). A meta-analysis revealed that a benefit of non-pharmacological treatments is their multi-modal effects that can reduce not only pain but also fatigue and nonrestorative sleep associated with fibromyalgia (41). The effect of TENS on sleep disturbance is moderate (Active-TENS vs. Placebo-TENS, Cohen's *d* = 0.7) and is within the range of other effective pharmacological and non-pharmacological treatments as reported in the meta-analysis by Perrot et al. (Sleep disturbance, Cohen's d = amitriptyline: 0.7, sodium oxybate: 0.5, cognitive behavioral therapy: 0.4, magnetic cerebral stimulation: 0.5) (41). Although the clinical significance of improvements in restorative sleep during the blinded and unblinded phases of this study is undetermined, the findings support that TENS is a low-cost adjunct to care for women with fibromyalgia who report nonrestorative sleep.

### Limitations

A limitation in the generalizability of these findings is that the eligibility criteria only included women with fibromyalgia who met the 1990 ACR criteria. As a secondary measure, study personnel also assessed the 2010 ACR criteria for fibromyalgia. While most participants met both diagnostic criteria, 3/301 participants did not meet the 2010 criteria. The effect of TENS on sleep in a more heterogeneous sample of men and women with fibromyalgia who meet the 2016 ACR criteria for fibromyalgia is unknown.

Another limitation of the study is the inability to achieve sufficient blinding in the Active-TENS group when TENS is used at an adequate intensity (strong and comfortable) to produce analgesia (29, 42). This potential source of bias is challenging to avoid despite the methods utilized in this study to maximize blinding. Correctly guessing treatment allocation may affect TENS use and contribute to the correlation between greater TENS use and a greater improvement in sleep.

The lack of a detected effect on actigraph-measured sleep could be due to a lack of efficacy or a lack of validity. While actigraph-measured sleep has demonstrated concurrent validity with polysomnography (32), it remains unknown how actigraph-measured sleep compares with polysomnography-measured sleep in women with fibromyalgia. Since this study did not assess polysomnography, we can only comment on poor concurrent validity with self-report measures in women with fibromyalgia.

## Conclusions

The findings suggest that the PROMIS sleep short forms are reliable and sensitive to changes in sleep in women with fibromyalgia. This is may be due to better precision and/or accurate measurement of particular aspects of restorative sleep than the PSQI. A month of daily TENS use improved self-reported sleep in women with fibromyalgia by a statistically significant amount, although the clinical relevance of this improvement is unclear. TENS may influence self-reported sleep by reducing pain, which allows women with fibromyalgia to improve daytime function and sleep quality at night. TENS is a multi-modal, non-pharmacological treatment option for women with fibromyalgia who report nonrestorative sleep.

## Data Availability Statement

The original contributions presented in the study are included in the article/Supplementary Material, further inquiries can be directed to the corresponding authors.

## Ethics Statement

The studies involving human participants were reviewed and approved by the University of Iowa Institutional Review Board and Vanderbilt University Institutional Review Board. The patients/participants provided their written informed consent to participate in this study.

## Author Contributions

BR, DD, CV, MZ, LC, and KS contributed to the conception and design of the study. RC, DD, CV, KG, and JW contributed to data acquisition. RC, BR, MZ, LC, and KS contributed to data analysis and interpretation. All the authors were involved in drafting the article or revising it critically for important intellectual content, and all approved the final version to be published.

## Conflict of Interest

KS serves as a consultant for Pfizer Consumer Health and Novartis Consumer Healthcare/GSK Consumer Healthcare, had an active grant from the American Pain Society/Pfizer, and receives royalties from IASP Press. The remaining authors declare that the research was conducted in the absence of any commercial or financial relationships that could be construed as a potential conflict of interest.
