# Supplementary material for: Test–Retest Reliability and Responsiveness of PROMIS Sleep Short Forms Within an RCT in Women With Fibromyalgia
Source: Front Pain Res (Lausanne). 2021 Jun 8;2:682072. doi: 10.3389/fpain.2021.682072 (PMC8915631; doi:10.3389/fpain.2021.682072)
Supplement: Supplementary file 1 [file Table_1.DOCX]

**Supplement 1**. Mean (standard error) for PROMIS measures by time. Treatment group (Trt), Time, and Trt*Time interactions effects for PROMIS measures with the data at visit timepoints provided as mean (standard deviation). The group mean differences (95% confidence interval) for Active- versus Placebo- and versus No-TENS groups are provided over the 4-weeks of randomized, blinded treatment (0-weeks to 4-weeks) and over the 2^nd^ month of non-randomized, all active TENS treatment (0-weeks to 8-weeks).

|  | Active | Placebo | No-TENS | Trt |  |
| --- | --- | --- | --- | --- | --- |
| **PROMIS Sleep Disturbance** | | | | | |
| 0-weeks | 61.8 (0.8) | 60.2 (0.8) | 60.7 (0.8) | p=0.37 |  |
| 4-weeks | 59.9 (0.9) | 58.9 (0.9) | 60.6 (0.9) | p=0.37 |  |
| 8-weeks | 59.3 (1.0) | 56.8 (1.0) | 58.1 (1.0) |  |  |
| 0-weeks to 4-weeks, Trt*Time Interaction, p=0.08 | | | | | |
| Change | -1.9 (-3.6, -0.3) | -1.3 (-3.0, 0.3) | -0.1 (-1.6, 1.5) |  |  |
| Time | **p=0.010** | p=0.18 | p>0.99 |  |  |
| 0-weeks to 8-weeks | | | | | |
| Change | -2.5 (-4.5, -0.6) | -3.5 (-5.5, -1.4) | -2.6 (-4.5,-0.7) |  |  |
| Time | **p=0.005** | **p<0.001** | **p=0.002** |  |  |
| **PROMIS Sleep-Related Impairment** | | |  |  | |
| 0-weeks | 64.1 (0.7) | 61.7 (0.7) | 62.1 (0.7) | **p=0.040** |  |
| 4-weeks | 61.1 (0.8) | 60.5 (0.8) | 62.0 (0.8) | p=0.43 |  |
| 8-weeks | 61.7 (0.9) | 58.1 (0.9) | 59.4 (0.9) |  |  |
| 0-weeks to 4-weeks, Trt*Time Interaction effect, **p=0.003*** | | | | | |
| Change | **-**3.0 (-4.6, -1.4) | -1.2 (-2.8, 0.4) | -0.2 (-1.7, 1.4) |  |  |
| Time | **p<0.001** | p=0.28 | p>0.99 |  |  |
| 0-weeks to 8-weeks | | | | |  |
| Change | -2.4 (-4.3, -0.5) | -3.6 (-5.5, -1.6) | -2.7 (-4.6,-0.9) |  |  |
| Time | **p=0.006** | **p<0.001** | **p<0.001** |  |  |
| **PROMIS Fatigue** | | | | |  |
| 0-weeks | 65.3 (0.6) | 63.5 (0.6) | 64.5 (0.6) | p=0.09 |  |
| 4-weeks | 62.9 (0.7) | 62.3 (0.7) | 64.2 (0.7) | p=0.17 |  |
| 8-weeks | 62.4 (0.7) | 60.5 (0.8) | 61.8 (0.7) |  |  |
| 0-weeks to 4-weeks, Treatment*Time Interaction effect, **p=0.03**** | | | | | |
| Change | -2.4 (-3.9, -0.9) | -1.1 (-2.7, 0.4) | -0.3 (-1.8, 1.2) |  |  |
| Time | **p<0.001** | p=0.29 | p>0.99 |  |  |
| 0-weeks to 8-weeks | | | | |  |
| Change | -2.9 (-4.7, -1.0) | -2.9 (-4.8, -1.1) | -2.7 (-4.5,-0.9) |  |  |
| Time | **p<0.001** | **p<0.001** | **p<0.001** |  |  |

*** p=0.136 when resting pain included in statistical model**

**** p=0.505 with resting pain included in the statistical model**

**Supplement 2**. Treatment group (Trt), Time, and Trt*Time interactions effects for Actigraph-measured sleep and the Pittsburgh Sleep Quality Index (PSQI). Data presented as mean (standard deviation) or mean difference (95% Confidence Interval).

|  | Active TENS | Placebo TENS | No TENS |
| --- | --- | --- | --- |
|  | n=103 | n=99 | n=99 |
| **Actigraph- Sleep Efficiency** | |  |  |
| 0-weeks (Trt p=0.04) 89.0 (0.6) | | 87.2 (0.6) | 89.1 (0.6) |
| 4-weeks (Trt p=0.73) 88.1 (0.8) | | 87.2 (0.8) | 87.5 (0.8) |
| 8-weeks | 88.6 (0.8) | 87.5 (0.8) | 88.2 (0.8) |
| Change 0 to 4 weeks  (Trt*Time p=0.27) | -1.0 (-2.8, 0.9)  p=0.94 | 0.0 (-1.9, 1.9)  p>0.99 | -1.6 (-3.4, 0.2)  p=0.10 |
| Change 0 to 8 weeks | -0.4 (-2.3, 1.5)  p>0.99 | 0.3 (-1.6, 2.2)  p>0.99 | -1.0 (-2.8, 0.9)  p=0.84 |
| **Actigraph- Total Sleep Time** | |  |  |
| 0-weeks (Trt p=0.48) 408.9 (8.0) | | 420.8 (8.2) | 421.0 (8.2) |
| 4-weeks (Trt p=0.27) 412.2 (8.8) | | 415.7 (9.1) | 430.7 (8.4) |
| 8-weeks | 426.3 (9.3) | 425.5 (9.2) | 432.1 (8.9) |
| Change 0 to 4 weeks  (Trt*Time p=0.49) | 3.3 (-19.8, 26.4)  p>0.99 | -5.1 (-28.9, 18.7)  p>0.99 | 9.6 (-12.5, 31.7)  p>0.99 |
| Change 0 to 8 weeks | 17.4 (-6.9, 41.7)  p=0.33 | 4.7 (-19.5, 28.8)  p>0.99 | 11.0 (-12.5, 34.5)  p>0.99 |
| **PSQI Total** |  |  |  |
| 0-weeks (Trt p=0.38) 12.6 (0.4) | | 12.0 (0.4) | 11.9 (0.4) |
| 4-weeks  (Trt p=0.54) | 11.7 (0.4) | 11.1 (0.4) | 11.6 (0.4) |
| 8-weeks | 11.3 (0.4) | 10.4 (0.4) | 10.7 (0.4) |
| Change 0 to 4 weeks  (Trt*Time p=0.28) | -0.9 (-1.7, -0.1)  **p=0.03** | -0.9 (-1.7, -0.0)  **p=0.04** | -0.3 (-1.1, 0.5)  p>0.99 |
| Change 0 to 8 weeks | -1.2 (-2.1, -0.3)  **p=0.002** | -1.6 (-2.5, -0.7)  **p<0.001** | -1.2 (-2.1,-0.4)  **p=0.001** |

**Supplement 3**. Treatment (Trt), Trt*time, and Trt*Opioid interaction effects for self-reported sleep and fatigue as well as actigraph-measured sleep adjusted for the covariates of marital status and baseline FIQR.

|  | Group Mean Difference (95% CI) | |
| --- | --- | --- |
|  | Active v. Placebo | Active v. No TENS |
| **PROMIS sleep disturbance** | |  |
| 4-weeks  (Trt p=0.40) | 0.37 (-2.44, 3.18)  p=0.95 | -1.14 (-3.90, 1.62)  p=0.59 |
| Change 0 to 4 weeks  (Trt*Time p=0.08) | -0.58 (-2.67, 1.52)  p>0.99 | -1.87 (-3.91, 0.17)  p=0.09 |
| **PROMIS sleep impairment** | |  |
| 4-weeks (Trt p=0.23) | 0.51 (-2.96, 1.95)  p=0.88 | 1.70 (-4.11, 0.71)  p=0.22 |
| Change 0- to 4 weeks  (Trt*Time **p=0.002**)  Trt*Opioid | -1.83 (-3.89, 0.23)  p=0.10 | -2.94 (-4.96, -0.93)  **p=0.002** |
| **PROMIS fatigue** |  |  |
| 4-weeks  (Trt p=0.04)* | -0.74 (-2.82, 1.34)  p=0.68 | -2.14 (-4.17, -0.11)  p=0.04* |
| Change 0 to 4 weeks  (Trt*Time **p=0.02**)  Trt*Opioid | -1.39 (-3.34, 0.55)  p=0.26 | -2.21 (-4.10, -0.32)  **p=0.02** |
| **PSQI total** |  |  |
| 4-weeks  (Trt p=0.73) | 0.13 (-1.16, 1.41)  p=0.97 | -0.29 (-1.55, 0.98)  p=0.86 |
| Change 0 to 4 weeks  (Trt*Time p=0.26) | 0.00 (-1.08, 1.09)  p>0.99 | -0.62 (-1.68, 0.44)  p=0.48 |
| **Actigraph- sleep efficiency** | |  |
| 4-weeks  (Trt p=0.27) | 1.84 (-0.91, 4.60)  p=0.26 | 1.29 (-1,35, 3.94)  p=0.48 |
| Change 0 to 4 weeks  (Trt*Time p=0.30) | -0.85 (-3.33, 1.63)  p>0.99 | 0.71 (-1.66, 3.08)  p>0.99 |
| **Actigraph- total sleep time** | |  |
| 4-weeks  (Trt p=0.40) | 3.4 (-26.9, 33.7)  p=0.96 | -12.3 (-41.3, 16.6)  p=0.58 |
| Change 0 to 4 weeks  (Trt*Time p=0.44) | 9.6 (-20.9, 40.0)  p>0.99 | -6.2 (-35.5, 23.1)  p>0.99 |

* Indicates that statistical significance differs from un-adjusted comparisons.
